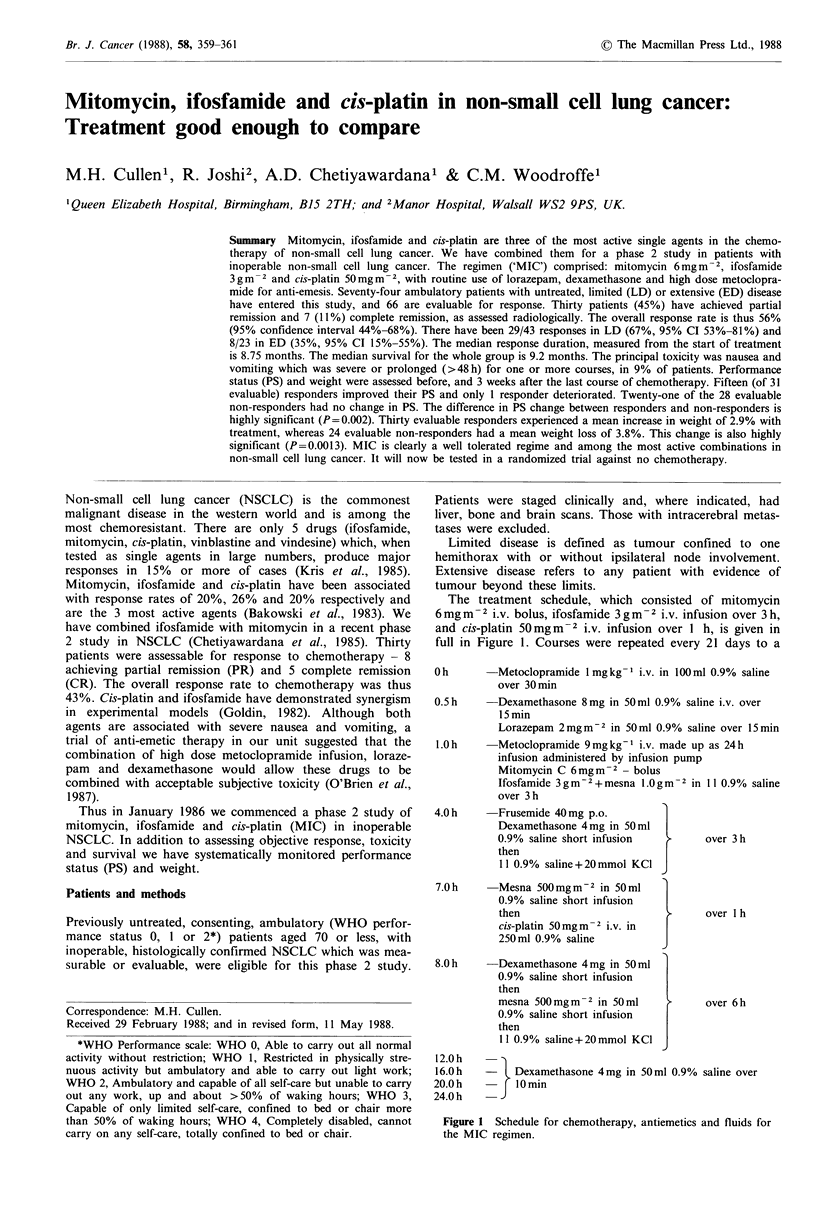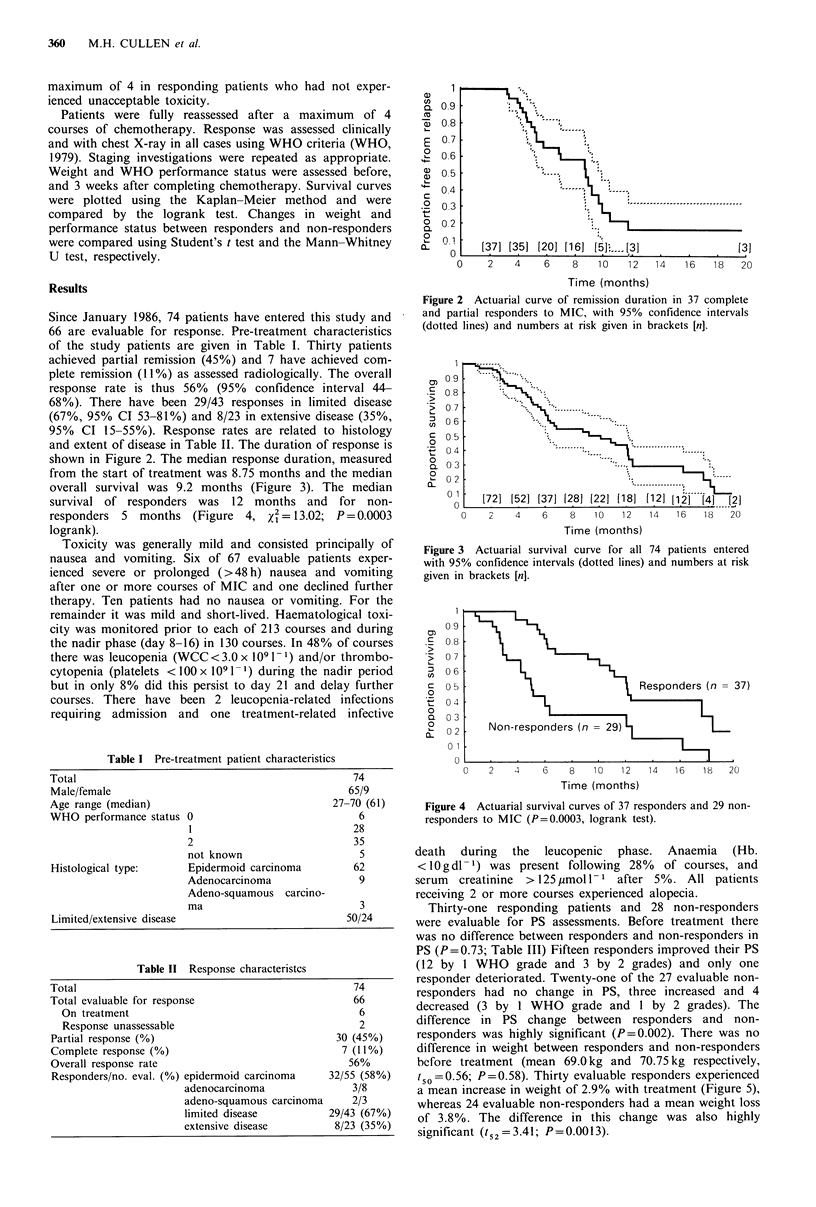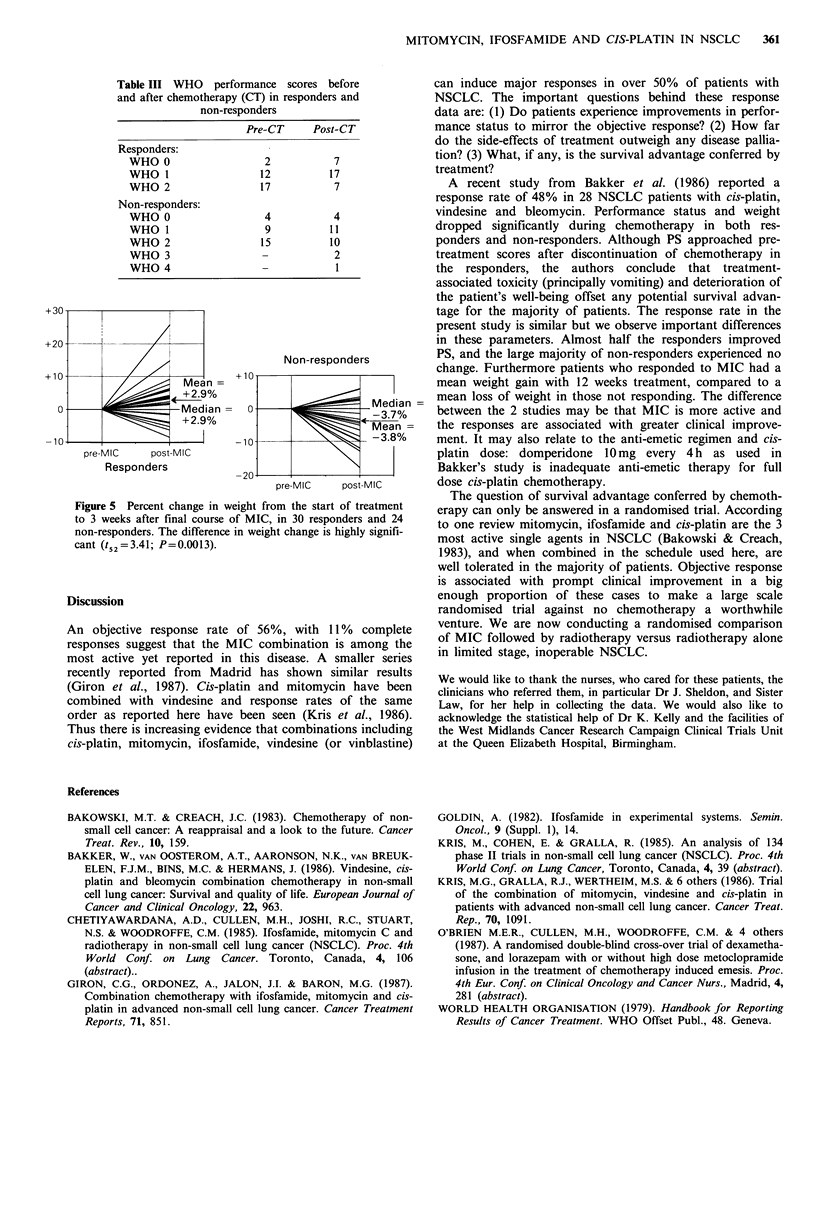# Mitomycin, ifosfamide and cis-platin in non-small cell lung cancer: treatment good enough to compare.

**DOI:** 10.1038/bjc.1988.219

**Published:** 1988-09

**Authors:** M. H. Cullen, R. Joshi, A. D. Chetiyawardana, C. M. Woodroffe

**Affiliations:** Queen Elizabeth Hospital, Birmingham, UK.

## Abstract

Mitomycin, ifosfamide and cis-platin are three of the most active single agents in the chemotherapy of non-small cell lung cancer. We have combined them for a phase 2 study in patients with inoperable non-small cell lung cancer. The regimen ('MIC') comprised: mitomycin 6 mg m-2, ifosfamide 3 g m-2 and cis-platin 50 mg m-2, with routine use of lorazepam, dexamethasone and high dose metoclopramide for anti-emesis. Seventy-four ambulatory patients with untreated, limited (LD) or extensive (ED) disease have entered this study, and 66 are evaluable for response. Thirty patients (45%) have achieved partial remission and 7 (11%) complete remission, as assessed radiologically. The overall response rate is thus 56% (95% confidence interval 44%-68%). There have been 29/43 responses in LD (67%, 95% CI 53%-81%) and 8/23 in ED (35%, 95% CI 15%-55%). The median response duration, measured from the start of treatment is 8.75 months. The median survival for the whole group is 9.2 months. The principal toxicity was nausea and vomiting which was severe or prolonged (greater than 48 h) for one or more courses, in 9% of patients. Performance status (PS) and weight were assessed before, and 3 weeks after the last course of chemotherapy. Fifteen (of 31 evaluable) responders improved their PS and only 1 responder deteriorated. Twenty-one of the 28 evaluable non-responders had no change in PS. The difference in PS change between responders and non-responders is highly significant (P = 0.002). Thirty evaluable responders experienced a mean increase in weight of 2.9% with treatment, whereas 24 evaluable non-responders had a mean weight loss of 3.8%. This change is also highly significant (P = 0.0013). MIC is clearly a well tolerated regime and among the most active combinations in non-small cell lung cancer. It will now be tested in a randomized trial against no chemotherapy.


*Br. J. Cancer* (1988), **58**, 359–361 © The Macmillan Press Ltd., 1988

### Mitomycin, ifosfamide and *cis*-platin in non-small cell lung cancer: Treatment good enough to compare

M.H. Cullen[1], R. Joshi[2], A.D. Chetiyawardana[1] & C.M. Woodroffe[1]

[1]*Queen Elizabeth Hospital, Birmingham, B15 2TH; and* [2]*Manor Hospital, Walsall WS2 9PS, UK.*

**Summary** Mitomycin, ifosfamide and *cis*-platin are three of the most active single agents in the chemotherapy of non-small cell lung cancer. We have combined them for a phase 2 study in patients with inoperable non-small cell lung cancer. The regimen ('MIC') comprised: mitomycin $6 mg m^{-2}$, ifosfamide $3 g m^{-2}$ and *cis*-platin $50 mg m^{-2}$, with routine use of lorazepam, dexamethasone and high dose metoclopramide for anti-emesis. Seventy-four ambulatory patients with untreated, limited (LD) or extensive (ED) disease have entered this study, and 66 are evaluable for response. Thirty patients (45%) have achieved partial remission and 7 (11%) complete remission, as assessed radiologically. The overall response rate is thus 56% (95% confidence interval 44%–68%). There have been 29/43 responses in LD (67%, 95% CI 53%–81%) and 8/23 in ED (35%, 95% CI 15%–55%). The median response duration, measured from the start of treatment is 8.75 months. The median survival for the whole group is 9.2 months. The principal toxicity was nausea and vomiting which was severe or prolonged (>48 h) for one or more courses, in 9% of patients. Performance status (PS) and weight were assessed before, and 3 weeks after the last course of chemotherapy. Fifteen (of 31 evaluable) responders improved their PS and only 1 responder deteriorated. Twenty-one of the 28 evaluable non-responders had no change in PS. The difference in PS change between responders and non-responders is highly significant ($P=0.002$). Thirty evaluable responders experienced a mean increase in weight of 2.9% with treatment, whereas 24 evaluable non-responders had a mean weight loss of 3.8%. This change is also highly significant ($P=0.0013$). MIC is clearly a well tolerated regime and among the most active combinations in non-small cell lung cancer. It will now be tested in a randomized trial against no chemotherapy.

Non-small cell lung cancer (NSCLC) is the commonest malignant disease in the western world and is among the most chemoresistant. There are only 5 drugs (ifosfamide, mitomycin, *cis*-platin, vinblastine and vindesine) which, when tested as single agents in large numbers, produce major responses in 15% or more of cases (Kris *et al.*, 1985). Mitomycin, ifosfamide and *cis*-platin have been associated with response rates of 20%, 26% and 20% respectively and are the 3 most active agents (Bakowski *et al.*, 1983). We have combined ifosfamide with mitomycin in a recent phase 2 study in NSCLC (Chetiyawardana *et al.*, 1985). Thirty patients were assessable for response to chemotherapy – 8 achieving partial remission (PR) and 5 complete remission (CR). The overall response rate to chemotherapy was thus 43%. *Cis*-platin and ifosfamide have demonstrated synergism in experimental models (Goldin, 1982). Although both agents are associated with severe nausea and vomiting, a trial of anti-emetic therapy in our unit suggested that the combination of high dose metoclopramide infusion, lorazepam and dexamethasone would allow these drugs to be combined with acceptable subjective toxicity (O'Brien *et al.*, 1987).

Thus in January 1986 we commenced a phase 2 study of mitomycin, ifosfamide and *cis*-platin (MIC) in inoperable NSCLC. In addition to assessing objective response, toxicity and survival we have systematically monitored performance status (PS) and weight.

### Patients and methods

Previously untreated, consenting, ambulatory (WHO performance status 0, 1 or 2*) patients aged 70 or less, with inoperable, histologically confirmed NSCLC which was measurable or evaluable, were eligible for this phase 2 study. Patients were staged clinically and, where indicated, had liver, bone and brain scans. Those with intracerebral metastases were excluded.

Limited disease is defined as tumour confined to one hemithorax with or without ipsilateral node involvement. Extensive disease refers to any patient with evidence of tumour beyond these limits.

The treatment schedule, which consisted of mitomycin $6 mg m^{-2}$ i.v. bolus, ifosfamide $3 g m^{-2}$ i.v. infusion over 3 h, and *cis*-platin $50 mg m^{-2}$ i.v. infusion over 1 h, is given in full in Figure 1. Courses were repeated every 21 days to a

| | | |
|---|---|---|
| 0 h | —Metoclopramide $1 mg kg^{-1}$ i.v. in 100 ml 0.9% saline over 30 min | |
| 0.5 h | —Dexamethasone 8 mg in 50 ml 0.9% saline i.v. over 15 min<br>Lorazepam $2 mg m^{-2}$ in 50 ml 0.9% saline over 15 min | |
| 1.0 h | —Metoclopramide $9 mg kg^{-1}$ i.v. made up as 24 h infusion administered by infusion pump<br>Mitomycin C $6 mg m^{-2}$ – bolus<br>Ifosfamide $3 g m^{-2}$ + mesna $1.0 g m^{-2}$ in 1 l 0.9% saline over 3 h | |
| 4.0 h | —Frusemide 40 mg p.o.<br>Dexamethasone 4 mg in 50 ml 0.9% saline short infusion then<br>1 l 0.9% saline + 20 mmol KCl | over 3 h |
| 7.0 h | —Mesna $500 mg m^{-2}$ in 50 ml 0.9% saline short infusion then<br>*cis*-platin $50 mg m^{-2}$ i.v. in 250 ml 0.9% saline | over 1 h |
| 8.0 h | —Dexamethasone 4 mg in 50 ml 0.9% saline short infusion then<br>mesna $500 mg m^{-2}$ in 50 ml 0.9% saline short infusion then<br>1 l 0.9% saline + 20 mmol KCl | over 6 h |
| 12.0 h<br>16.0 h<br>20.0 h<br>24.0 h | — Dexamethasone 4 mg in 50 ml 0.9% saline over 10 min | |

**Figure 1** Schedule for chemotherapy, antiemetics and fluids for the MIC regimen.

Correspondence: M.H. Cullen.
Received 29 February 1988; and in revised form, 11 May 1988.

*WHO Performance scale: WHO 0, Able to carry out all normal activity without restriction; WHO 1, Restricted in physically strenuous activity but ambulatory and able to carry out light work; WHO 2, Ambulatory and capable of all self-care but unable to carry out any work, up and about >50% of waking hours; WHO 3, Capable of only limited self-care, confined to bed or chair more than 50% of waking hours; WHO 4, Completely disabled, cannot carry on any self-care, totally confined to bed or chair.

maximum of 4 in responding patients who had not experienced unacceptable toxicity.

Patients were fully reassessed after a maximum of 4 courses of chemotherapy. Response was assessed clinically and with chest X-ray in all cases using WHO criteria (WHO, 1979). Staging investigations were repeated as appropriate. Weight and WHO performance status were assessed before, and 3 weeks after completing chemotherapy. Survival curves were plotted using the Kaplan–Meier method and were compared by the logrank test. Changes in weight and performance status between responders and non-responders were compared using Student's *t* test and the Mann–Whitney U test, respectively.

### Results

Since January 1986, 74 patients have entered this study and 66 are evaluable for response. Pre-treatment characteristics of the study patients are given in Table I. Thirty patients achieved partial remission (45%) and 7 have achieved complete remission (11%) as assessed radiologically. The overall response rate is thus 56% (95% confidence interval 44–68%). There have been 29/43 responses in limited disease (67%, 95% CI 53–81%) and 8/23 in extensive disease (35%, 95% CI 15–55%). Response rates are related to histology and extent of disease in Table II. The duration of response is shown in Figure 2. The median response duration, measured from the start of treatment was 8.75 months and the median overall survival was 9.2 months (Figure 3). The median survival of responders was 12 months and for non-responders 5 months (Figure 4, $\chi^2_1 = 13.02$; $P = 0.0003$ logrank).

Toxicity was generally mild and consisted principally of nausea and vomiting. Six of 67 evaluable patients experienced severe or prolonged (>48 h) nausea and vomiting after one or more courses of MIC and one declined further therapy. Ten patients had no nausea or vomiting. For the remainder it was mild and short-lived. Haematological toxicity was monitored prior to each of 213 courses and during the nadir phase (day 8–16) in 130 courses. In 48% of courses there was leucopenia (WCC $< 3.0 \times 10^9 l^{-1}$) and/or thrombocytopenia (platelets $< 100 \times 10^9 l^{-1}$) during the nadir period but in only 8% did this persist to day 21 and delay further courses. There have been 2 leucopenia-related infections requiring admission and one treatment-related infective

**Table I** Pre-treatment patient characteristics

| | | |
|---|---|---|
| Total | | 74 |
| Male/female | | 65/9 |
| Age range (median) | | 27–70 (61) |
| WHO performance status | 0 | 6 |
| | 1 | 28 |
| | 2 | 35 |
| | not known | 5 |
| Histological type: | Epidermoid carcinoma | 62 |
| | Adenocarcinoma | 9 |
| | Adeno-squamous carcinoma | 3 |
| Limited/extensive disease | | 50/24 |

**Table II** Response characteristcs

| | | |
|---|---|---|
| Total | | 74 |
| Total evaluable for response | | 66 |
| On treatment | | 6 |
| Response unassessable | | 2 |
| Partial response (%) | | 30 (45%) |
| Complete response (%) | | 7 (11%) |
| Overall response rate | | 56% |
| Responders/no. eval. (%) | epidermoid carcinoma | 32/55 (58%) |
| | adenocarcinoma | 3/8 |
| | adeno-squamous carcinoma | 2/3 |
| | limited disease | 29/43 (67%) |
| | extensive disease | 8/23 (35%) |

**Figure 2** Actuarial curve of remission duration in 37 complete and partial responders to MIC, with 95% confidence intervals (dotted lines) and numbers at risk given in brackets [*n*].

**Figure 3** Actuarial survival curve for all 74 patients entered with 95% confidence intervals (dotted lines) and numbers at risk given in brackets [*n*].

**Figure 4** Actuarial survival curves of 37 responders and 29 non-responders to MIC ($P = 0.0003$, logrank test).

death during the leucopenic phase. Anaemia (Hb. $< 10 \text{ g dl}^{-1}$) was present following 28% of courses, and serum creatinine $> 125 \mu\text{mol l}^{-1}$ after 5%. All patients receiving 2 or more courses experienced alopecia.

Thirty-one responding patients and 28 non-responders were evaluable for PS assessments. Before treatment there was no difference between responders and non-responders in PS ($P = 0.73$; Table III) Fifteen responders improved their PS (12 by 1 WHO grade and 3 by 2 grades) and only one responder deteriorated. Twenty-one of the 27 evaluable non-responders had no change in PS, three increased and 4 decreased (3 by 1 WHO grade and 1 by 2 grades). The difference in PS change between responders and non-responders was highly significant ($P = 0.002$). There was no difference in weight between responders and non-responders before treatment (mean 69.0 kg and 70.75 kg respectively, $t_{50} = 0.56$; $P = 0.58$). Thirty evaluable responders experienced a mean increase in weight of 2.9% with treatment (Figure 5), whereas 24 evaluable non-responders had a mean weight loss of 3.8%. The difference in this change was also highly significant ($t_{52} = 3.41$; $P = 0.0013$).

**Table III** WHO performance scores before and after chemotherapy (CT) in responders and non-responders

| | *Pre-CT* | *Post-CT* |
|---|---|---|
| Responders: | | |
| WHO 0 | 2 | 7 |
| WHO 1 | 12 | 17 |
| WHO 2 | 17 | 7 |
| Non-responders: | | |
| WHO 0 | 4 | 4 |
| WHO 1 | 9 | 11 |
| WHO 2 | 15 | 10 |
| WHO 3 | – | 2 |
| WHO 4 | – | 1 |

**Figure 5** Percent change in weight from the start of treatment to 3 weeks after final course of MIC, in 30 responders and 24 non-responders. The difference in weight change is highly significant ($t_{52}=3.41$; $P=0.0013$).

### Discussion

An objective response rate of 56%, with 11% complete responses suggest that the MIC combination is among the most active yet reported in this disease. A smaller series recently reported from Madrid has shown similar results (Giron *et al.*, 1987). *Cis*-platin and mitomycin have been combined with vindesine and response rates of the same order as reported here have been seen (Kris *et al.*, 1986). Thus there is increasing evidence that combinations including *cis*-platin, mitomycin, ifosfamide, vindesine (or vinblastine) can induce major responses in over 50% of patients with NSCLC. The important questions behind these response data are: (1) Do patients experience improvements in performance status to mirror the objective response? (2) How far do the side-effects of treatment outweigh any disease palliation? (3) What, if any, is the survival advantage conferred by treatment?

A recent study from Bakker *et al.* (1986) reported a response rate of 48% in 28 NSCLC patients with *cis*-platin, vindesine and bleomycin. Performance status and weight dropped significantly during chemotherapy in both responders and non-responders. Although PS approached pretreatment scores after discontinuation of chemotherapy in the responders, the authors conclude that treatment-associated toxicity (principally vomiting) and deterioration of the patient's well-being offset any potential survival advantage for the majority of patients. The response rate in the present study is similar but we observe important differences in these parameters. Almost half the responders improved PS, and the large majority of non-responders experienced no change. Furthermore patients who responded to MIC had a mean weight gain with 12 weeks treatment, compared to a mean loss of weight in those not responding. The difference between the 2 studies may be that MIC is more active and the responses are associated with greater clinical improvement. It may also relate to the anti-emetic regimen and *cis*-platin dose: domperidone 10 mg every 4 h as used in Bakker's study is inadequate anti-emetic therapy for full dose *cis*-platin chemotherapy.

The question of survival advantage conferred by chemotherapy can only be answered in a randomised trial. According to one review mitomycin, ifosfamide and *cis*-platin are the 3 most active single agents in NSCLC (Bakowski & Creach, 1983), and when combined in the schedule used here, are well tolerated in the majority of patients. Objective response is associated with prompt clinical improvement in a big enough proportion of these cases to make a large scale randomised trial against no chemotherapy a worthwhile venture. We are now conducting a randomised comparison of MIC followed by radiotherapy versus radiotherapy alone in limited stage, inoperable NSCLC.

We would like to thank the nurses, who cared for these patients, the clinicians who referred them, in particular Dr J. Sheldon, and Sister Law, for her help in collecting the data. We would also like to acknowledge the statistical help of Dr K. Kelly and the facilities of the West Midlands Cancer Research Campaign Clinical Trials Unit at the Queen Elizabeth Hospital, Birmingham.

### References

BAKOWSKI, M.T. & CREACH, J.C. (1983). Chemotherapy of non-small cell cancer: A reappraisal and a look to the future. *Cancer Treat. Rev.*, **10**, 159.

BAKKER, W., VAN OOSTEROM, A.T., AARONSON, N.K., VAN BREUKELEN, F.J.M., BINS, M.C. & HERMANS, J. (1986). Vindesine, *cis*-platin and bleomycin combination chemotherapy in non-small cell lung cancer: Survival and quality of life. *European Journal of Cancer and Clinical Oncology*, **22**, 963.

CHETIYAWARDANA, A.D., CULLEN, M.H., JOSHI, R.C., STUART, N.S. & WOODROFFE, C.M. (1985). Ifosfamide, mitomycin C and radiotherapy in non-small cell lung cancer (NSCLC). *Proc. 4th World Conf. on Lung Cancer.* Toronto, Canada, **4**, 106 (*abstract*)..

GIRON, C.G., ORDONEZ, A., JALON, J.I. & BARON, M.G. (1987). Combination chemotherapy with ifosfamide, mitomycin and *cis*-platin in advanced non-small cell lung cancer. *Cancer Treatment Reports*, **71**, 851.

GOLDIN, A. (1982). Ifosfamide in experimental systems. *Semin. Oncol.*, **9** (Suppl. 1), 14.

KRIS, M., COHEN, E. & GRALLA, R. (1985). An analysis of 134 phase II trials in non-small cell lung cancer (NSCLC). *Proc. 4th World Conf. on Lung Cancer*, Toronto, Canada, **4**, 39 (*abstract*).

KRIS, M.G., GRALLA, R.J., WERTHEIM, M.S. & 6 others (1986). Trial of the combination of mitomycin, vindesine and *cis*-platin in patients with advanced non-small cell lung cancer. *Cancer Treat. Rep.*, **70**, 1091.

O'BRIEN M.E.R., CULLEN, M.H., WOODROFFE, C.M. & 4 others (1987). A randomised double-blind cross-over trial of dexamethasone, and lorazepam with or without high dose metoclopramide infusion in the treatment of chemotherapy induced emesis. *Proc. 4th Eur. Conf. on Clinical Oncology and Cancer Nurs.*, Madrid, **4**, 281 (*abstract*).

WORLD HEALTH ORGANISATION (1979). *Handbook for Reporting Results of Cancer Treatment.* WHO Offset Publ., 48. Geneva.